# Variability in Refractive Measurements in Children with Autism Spectrum Disorder Under Cycloplegic and Non-Cycloplegic Conditions: Influence of Measurement Method

**DOI:** 10.3390/jcm15207889

**Published:** 2026-10-13

**Authors:** Monika Modrzejewska, Karolina Skonieczna-Żydecka, Anna Modrzejewska, Oliwia Zdanowska, Piotr Połubiński, Wiktoria Bosy-Gąsior, Wilhelm Grzesiak

**Affiliations:** 1 2nd Department of Ophthalmology, Pomeranian Medical University in Szczecin, 70-111 Szczecin, Poland; 2 Department of Human Nutrition and Metabolomics, Pomeranian Medical University in Szczecin, 71-460 Szczecin, Poland; 3 Department of Ophthalmology, Independent Public Healthcare Institution of the Ministry of the Interior and Administration, 70-382 Szczecin, Poland; 4 Department of Biostatistics, Bioinformatics and Animal Research Methods, West Pomeranian University of Technology in Szczecin, 71-310 Szczecin, Poland

**Keywords:** autism, refractive errors, autorefractometer, accommodation, Plusoptix, Retinomax, tropicamide

## Abstract

**Purpose:** To compare refractive measurements obtained with Retinomax K-plus 3 and Plusoptix A09 V in children with autism spectrum disorder (ASD) before and after cycloplegia and to assess inter-device agreement. **Methods:** This retrospective pilot study included 23 children with ASD (mean age 6.54 ± 2.83 years), of whom 14 underwent examination with two autorefractometers (Retinomax K-plus 3 and Plusoptix A09 V) before and after cycloplegia induced with 1% tropicamide. Direct inter-device comparisons were restricted to the 14 children with paired measurements and were performed using the Wilcoxon signed-rank test, with matched-pairs rank-biserial correlations reported as effect sizes. Agreement in spherical equivalent (SE) was assessed using Bland–Altman analysis. Cycloplegia-induced refractive shifts were compared between devices. Secondary comparisons with controls without documented neurodevelopmental disorders (*n* = 27) were restricted to Retinomax SE measurements and performed using analysis of covariance (ANCOVA), with age included as a continuous covariate. **Results:** Before cycloplegia, Retinomax showed significantly more myopic SE values than Plusoptix in both eyes (right eye [OD]: −1.21 ± 1.25 vs. 0.91 ± 0.67 D; left eye [OS]: −1.22 ± 1.15 vs. 0.77 ± 0.51 D; both *p* < 0.00417). Significant inter-device differences in SE persisted after cycloplegia (OD: 0.63 ± 0.94 vs. 2.00 ± 0.63 D; OS: 0.46 ± 0.96 vs. 1.98 ± 0.63 D; both *p* < 0.001). Bland–Altman analysis showed a systematic negative bias of Retinomax relative to Plusoptix, which decreased after cycloplegia but persisted (before: −2.12 D for OD and −1.99 D for OS; after: −1.37 D and −1.52 D, respectively). Cycloplegia-induced shifts were greater with Retinomax; however, the between-device differences were not statistically significant after Bonferroni correction. In secondary analyses, age-adjusted Retinomax SE remained significantly lower in the ASD group than in controls without documented neurodevelopmental disorders before and after cycloplegia (all *p* < 0.001). **Conclusions:** In this exploratory cohort of children with ASD, Retinomax measurements were associated with more myopic SE values than Plusoptix, particularly before cycloplegia. The inter-device bias decreased but persisted after cycloplegia. This reduction is compatible with a contribution of accommodation; however, the persistence of the bias after cycloplegia indicates that device-specific measurement characteristics should also be considered. Refractive measurements obtained with the two autorefractometers should therefore not be assumed to be directly interchangeable. These findings underscore the importance of cycloplegic control and careful interpretation of autorefractor measurements in children with ASD. Larger prospective studies are warranted.

## 1. Introduction

Autism spectrum disorder (ASD) is a neurodevelopmental condition characterized by persistent deficits in social communication and social interaction across multiple contexts, together with restricted, repetitive patterns of behavior, interests, or activities. Symptoms must be present in the early developmental period, cause clinically significant impairment in social, occupational, or other important areas of functioning, and are not better explained by intellectual disability or global developmental delay [1]. The global prevalence of ASD is estimated at approximately 0.6%, with increasing diagnostic rates reported in recent years [2]. Diagnosis is typically established between 36 and 120 months of age, with a mean age of approximately 55 months. ASD occurs more frequently in males, with a reported male-to-female ratio of approximately 3:1 [3].

The etiology of ASD remains unclear and is thought to involve a combination of genetic and environmental factors, including maternal immune activation and infections [4]. Proposed pathophysiological mechanisms include altered synaptic function, immune dysregulation, and disrupted brain connectivity [5]. Dysfunctions in sensory information processing, impaired integration across structural brain networks within the central nervous system, and alterations in neurotransmitter systems may also contribute to visual impairments [6].

Visual system disorders are estimated to affect 27–40% of individuals with ASD. The most frequently reported include refractive errors, strabismus, and amblyopia [7,8]. Significant refractive errors, depending on the source, are found in 12.4–68% of children with ASD [8,9,10]. Such heterogeneity likely reflects differences in study design, age distribution, level of cooperation, and the use or absence of cycloplegia during refractive assessment. Accurate refractive evaluation in children with ASD may be particularly challenging due to limited cooperation, sensory hypersensitivity, and potential alterations in accommodative response.

Visual impairments in children with ASD may further exacerbate communication difficulties and impair functional development in children with ASD [11]. Therefore, reliable refractive assessment is of clinical importance in this population. Autorefractors are frequently used in pediatric ophthalmology due to their rapid and objective measurement capabilities. However, device-related factors, such as working distance and monocular versus binocular measurement, may influence accommodative activation and thereby affect refractive outcomes, particularly under non-cycloplegic conditions.

Accommodation may contribute substantially to discrepancies between non-cycloplegic and cycloplegic refractive measurements in children. A myopic shift observed under non-cycloplegic conditions may reflect accommodative activity; however, the magnitude of such a shift may also be influenced by measurement variability and device-specific characteristics. Consequently, differences between pre- and post-cycloplegic measurements were treated in the present study as cycloplegia-induced refractive shifts rather than as a diagnostic criterion for pseudomyopia. Reliable refractive assessment in children with ASD therefore represents both a clinical and methodological challenge, particularly when autorefractors based on different measurement principles may yield systematically different refractive measurements.

The primary aim of the present study was to compare refractive measurements obtained with the Retinomax K-plus 3 and Plusoptix A09 V in the same children with ASD under non-cycloplegic and cycloplegic conditions, with particular emphasis on spherical equivalent (SE) and inter-device agreement. A secondary aim was to characterize cycloplegia-induced refractive shifts and to compare Retinomax measurements between children with ASD and controls without documented neurodevelopmental disorders, accounting for differences in age. Ophthalmologic findings in the ASD cohort were additionally described to provide clinical context for the refractive analyses.

## 2. Materials and Methods

### 2.1. Materials

This retrospective observational study was based on a review of archived medical records of children with a confirmed diagnosis of ASD who participated in sensory integration sessions at an early childhood development support center in Szczecin, Poland. All available medical records meeting the study eligibility criteria during the study period were included. No prospective patient involvement occurred as the study was based exclusively on previously collected clinical data. The ophthalmologic examinations were performed at the Pediatric Ward of the Second Department of Ophthalmology, University Clinical Hospital No. 2 of the Pomeranian Medical University in Szczecin, and the analyzed records covered examinations conducted between June 2020 and May 2021.

The ASD cohort comprised 23 children (7 girls and 16 boys), aged 2.5–12 years (mean age: 6.54 ± 2.83 years), whose medical records met the eligibility criteria. In participants with ASD, the diagnosis had been established by a psychiatrist before the ophthalmologic examination, according to International Classification of Diseases, 10th Revision (ICD-10) criteria within the category of pervasive developmental disorders (F84) [12]. Diagnostic information was obtained from archived clinical records and was not independently reassessed for the purposes of the present study. Information on the specific diagnostic instruments used to establish the diagnosis and on Diagnostic and Statistical Manual of Mental Disorders, Fifth Edition (DSM-5) severity levels was not available in the reviewed records. Inclusion criteria were a documented diagnosis of ASD, availability of refractive measurements obtained with Retinomax before and after cycloplegia, and sufficient clinical documentation for inclusion in the retrospective analysis. No a priori sample-size calculation was performed because of the retrospective and exploratory nature of the study; the study size was determined by the number of available medical records meeting the eligibility criteria during the study period.

Gestational age ranged from 35 to 41 weeks (mean: 39.5 ± 1.64 weeks), and birth weight ranged from 2.49 to 4.48 kg (mean: 3.50 ± 0.48 kg). Medical records were systematically reviewed to extract data on pregnancy, delivery, and perinatal history, as well as documented comorbidities and neurologic and ophthalmologic diagnoses established during early childhood. Visual acuity assessment using Snellen optotypes displayed on a CP-600P LCD chart panel (Frey S.J., Piaseczno, Poland) was feasible in 15 children (65%) because of communication limitations. Anterior segment examination using a Topcon SL-D4 slit lamp (Topcon Corporation, Tokyo, Japan) and indirect ophthalmoscopy were not consistently performed in all cases because of limited cooperation.

### 2.2. Refractive Assessment

Refractive values were extracted from medical records and included measurements from both eyes obtained before and after cycloplegia. Refraction was expressed as spherical diopters (Dsph), cylindrical diopters (Dcyl), and SE, all in diopters (D).

Refractive measurements were obtained using the Retinomax K-plus 3 autorefractometer (RIGHT MFG. Co., Ltd., Tokyo, Japan) and Plusoptix A09 photorefractor (Plusoptix GmbH, Nuremberg, Germany). Retinomax measurements were available for all 23 children with ASD. Measurements with both devices were attempted in all participants; however, complete Plusoptix measurements were obtained in 14 children. In the remaining nine children, a complete Plusoptix measurement could not be obtained because of limited cooperation during the examination. Consequently, direct inter-device comparisons were restricted to the 14 children for whom paired measurements from both devices were available. No imputation of unavailable measurements was performed; inter-device analyses were restricted to complete paired observations.

The examination sequence was standardized, with Plusoptix measurements always performed first, followed by Retinomax measurements. Plusoptix measurements were obtained at a greater working distance without contact with the patient, whereas Retinomax measurements required examination at close range. Refractive values recorded in the medical documentation were used for statistical analysis. Retinomax measurements were performed using the instrument’s automated fogging of the fixation target to reduce accommodation.

Cycloplegia was induced by administering 1% tropicamide eye drops (Tropicamidum WZF 1%; Warszawskie Zakłady Farmaceutyczne Polfa S.A., Warsaw, Poland) three times at 10-min intervals. Tropicamide was chosen because of its established safety profile, availability, and documented effectiveness in pediatric refractive assessment [13,14,15,16], although it provides weaker cycloplegia than cyclopentolate. Post-cycloplegic refractive measurements were performed 30 min after the final instillation. All refractive examinations were performed by the same ophthalmologist under consistent examination conditions.

The control group consisted of 27 children (mean age: 8.58 years) examined at the same ophthalmology center and during the same study period as the ASD cohort. Medical records were retrospectively selected from children undergoing ophthalmologic assessment at the Pediatric Ward of the Second Department of Ophthalmology, University Clinical Hospital No. 2 of the Pomeranian Medical University in Szczecin. The main inclusion criteria for the control group were the absence of a documented diagnosis of ASD or other neurodevelopmental disorders and the availability of refractive measurements obtained with Retinomax before and after cycloplegia. All available medical records meeting these criteria during the study period were included. Plusoptix measurements were not available for the control group; therefore, comparisons between children with ASD and controls without documented neurodevelopmental disorders were restricted to Retinomax measurements.

### 2.3. Statistical Analysis

Descriptive statistics were calculated for the analyzed variables and are presented as means and standard deviations (SD).

SE was calculated from Dsph and Dcyl using the formula:

$$SE=\;\frac{1}{2}\mathrm{Dcyl}+\mathrm{Dsph}$$

Normality of data distribution was assessed using the Shapiro–Wilk test. As several variables deviated from normality, paired comparisons between Plusoptix and Retinomax measurements within the ASD group (*n* = 14) were performed using the Wilcoxon signed-rank test. Within-group comparisons of refractive parameters before and after cycloplegia were also performed using the Wilcoxon signed-rank test.

The significance level was set at α = 0.05 (two-tailed). Given multiple comparisons across refractive parameters and eyes, Bonferroni correction was applied where appropriate. For comparisons presented in Table 1 (12 tests), the adjusted significance threshold was α = 0.0042. For Table 2 (6 tests), the adjusted threshold was α = 0.0083.

Comparisons between children with ASD and controls without documented neurodevelopmental disorders were performed as secondary analyses and were restricted to Retinomax measurements because Plusoptix measurements were not available for the control group. These analyses included all 23 children with ASD and 27 controls without documented neurodevelopmental disorders and focused on SE as the principal refractive outcome. Because the groups differed substantially in age, analysis of covariance (ANCOVA) was used to compare SE between groups while accounting for age as a continuous covariate [17]. Separate models were fitted for right eye (OD) and left eye (OS) before and after cycloplegia. In each model, diagnostic group (ASD vs. control) was included as a fixed factor and age as a continuous covariate.

The homogeneity-of-regression-slopes assumption was evaluated by initially fitting a model containing the Group × Age interaction. Because this interaction was not statistically significant in any of the four analyses, the interaction term was removed, and the final models included the main effects of Group and Age. Age-adjusted estimated marginal means with 95% confidence intervals (CIs) were calculated for each group, together with the adjusted between-group differences. Group effects were evaluated using the corresponding ANCOVA F tests.

For paired comparisons between Plusoptix and Retinomax, effect sizes were expressed as matched-pairs rank-biserial correlations (*r*_rb_) [18], calculated as:

$$r_{rb}=\;\frac{W^{+}-\;W^{-}}{W^{+}+W^{-}}$$

where *W*^+^ and *W*^−^ denote the sums of ranks corresponding to positive and negative paired differences, respectively. Paired differences were defined as Plusoptix minus Retinomax; therefore, positive values of *r*_rb_ indicate higher measurements obtained with Plusoptix, whereas negative values indicate higher measurements obtained with Retinomax. The coefficient ranges from −1 to +1, with values approaching either extreme indicating greater consistency in the direction of the paired differences.

Agreement between Retinomax and Plusoptix measurements was additionally assessed for SE using the Bland–Altman method [19] in the subgroup of 14 children with paired measurements from both devices. Separate analyses were performed for OD and OS before and after cycloplegia. For each child, the difference between measurements obtained with the two devices (Retinomax − Plusoptix) was plotted against the mean of the corresponding measurements. The mean difference between devices was interpreted as the systematic bias, and the 95% limits of agreement (LoA) were calculated as the mean difference ± 1.96 SD of the differences. This analysis was used to characterize both the direction and magnitude of systematic inter-device differences and the dispersion of individual differences, rather than to test for a statistically significant difference between devices.

Cycloplegia-induced refractive shifts were calculated for each device as the post-cycloplegic value minus the corresponding pre-cycloplegic value. In the paired subgroup of 14 children, these shifts were compared between Plusoptix and Retinomax using the Wilcoxon signed-rank test. Bonferroni correction was applied to the six comparisons (Dsph, Dcyl, and SE for OD and OS), resulting in an adjusted significance threshold of α = 0.00833.

All statistical tests were two-sided. Unless an adjusted significance threshold was specified for a predefined family of multiple comparisons, *p* < 0.05 was considered statistically significant. All statistical analyses were performed using Statistica, version 13 (TIBCO Software Inc., San Ramon, CA, USA).

## 3. Results

### 3.1. Clinical and Ophthalmologic Characteristics of the ASD Group

A total of 23 children with ASD were included, of whom 14 (61%) completed examinations with both autorefractometers. Visual acuity was assessed in 15 children (65%) with whom verbal interaction was possible, using Snellen charts. Best-corrected visual acuity was 5/5 in 11 children (73%). Two children (13%) had visual acuity of OD 5/8.33 and OS 5/5, one child (7%) had OD 5/6.25 and OS 5/8.33, and one child (7%) had OD 5/50 and OS 5/5. Ophthalmologic findings documented in the available records included exotropia in 2 children (9%), esotropia in 4 (17%), and cataract in 1 (4%).

### 3.2. Comparison Between Autorefractometers

Paired refractive measurements obtained with both Plusoptix and Retinomax were available for 14 children with ASD. Direct inter-device comparisons were therefore restricted to this subgroup (Table 1). Before cycloplegia, Plusoptix yielded significantly higher values than Retinomax for Dsph, Dcyl, and SE in both eyes, and all six differences remained statistically significant after Bonferroni correction (adjusted α = 0.00417). The differences were particularly consistent for SE, with matched-pairs rank-biserial correlations of 1.000 for OD and 0.981 for OS.

After cycloplegia, significant inter-device differences persisted for Dsph and SE in both eyes. Mean SE was 2.00 ± 0.63 D with Plusoptix compared with 0.63 ± 0.94 D with Retinomax for OD, and 1.98 ± 0.63 D compared with 0.46 ± 0.96 D, respectively, for OS (both *p* < 0.001). In contrast, differences in Dcyl did not remain statistically significant after Bonferroni correction (OD: *p* = 0.551; OS: *p* = 0.035).

To further examine whether the magnitude of the cycloplegia-induced refractive shift differed between the two devices, changes in refractive parameters were calculated as post-cycloplegic minus pre-cycloplegic values for each child in the paired subgroup (*n* = 14). These changes were then compared between Plusoptix and Retinomax using the Wilcoxon signed-rank test (Table 2).

Cycloplegia-induced SE shifts were numerically greater with Retinomax than with Plusoptix in both eyes (OD: 1.84 ± 1.11 vs. 1.09 ± 0.71 D; OS: 1.67 ± 1.19 vs. 1.20 ± 0.77 D). However, none of the six between-device comparisons of cycloplegia-induced shifts reached statistical significance after Bonferroni correction.

**Figure 1:**
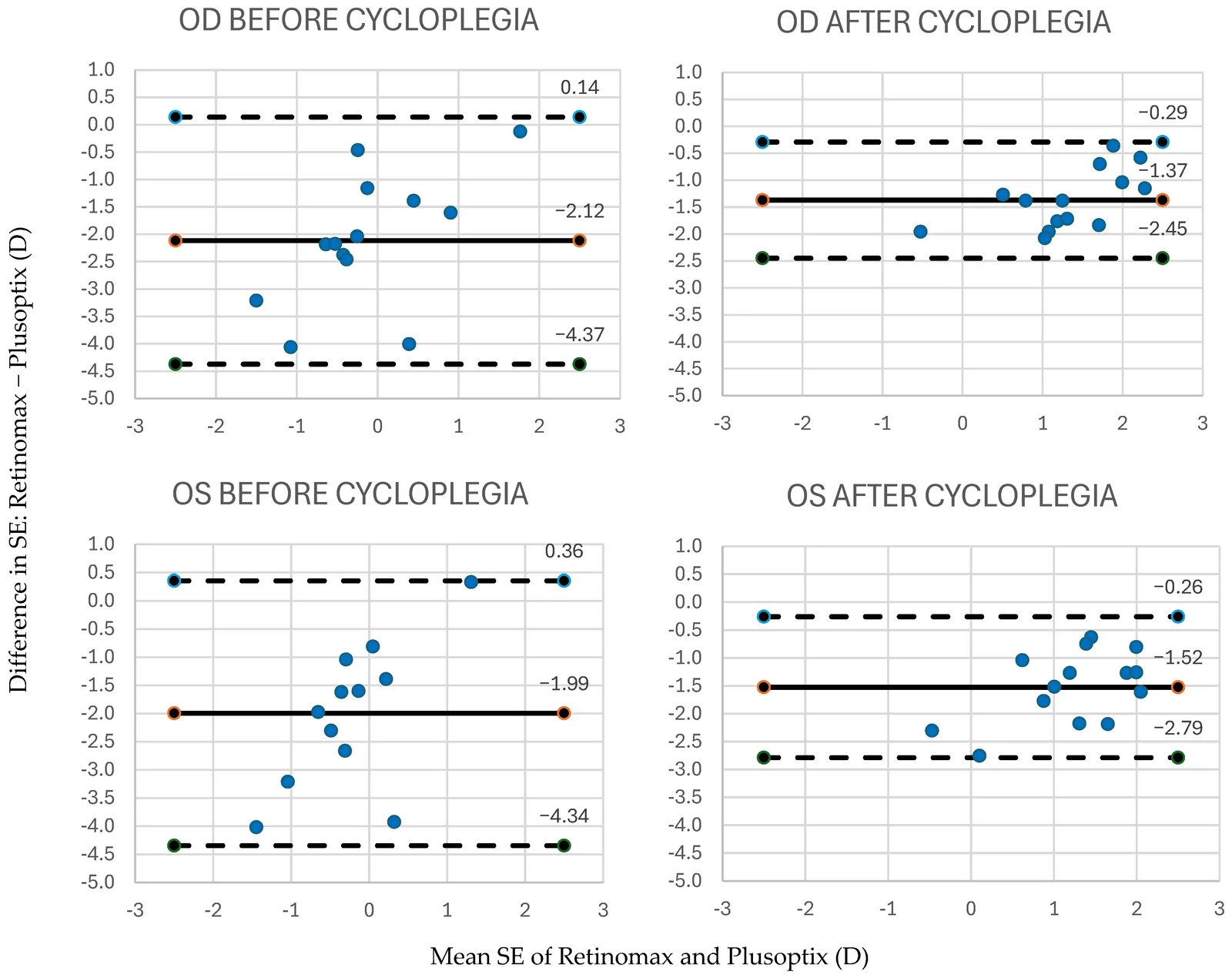
Bland–Altman plots of agreement between Retinomax and Plusoptix spherical equivalent (SE) measurements in children with autism spectrum disorder (ASD) (*n* = 14). Differences were calculated as Retinomax − Plusoptix. The solid horizontal line represents the mean difference (bias), and the dashed lines represent the 95% limits of agreement (mean difference ± 1.96 SD). OD—right eye; OS—left eye; D—diopters, SD—standard deviation.

Agreement between the two devices for SE was further assessed using Bland–Altman analysis (Figure 1). Before cycloplegia, the mean Retinomax–Plusoptix difference (bias) was −2.12 D for OD, with 95% LoA from −4.37 to 0.14 D, and −1.99 D for OS, with 95% LoA from −4.34 to 0.36 D. After cycloplegia, the magnitude of the bias was smaller but remained negative: −1.37 D for OD (95% LoA: −2.45 to −0.29 D) and −1.52 D for OS (95% LoA: −2.79 to −0.26 D). The limits of agreement were narrower after cycloplegia than before cycloplegia.

### 3.3. Secondary Comparison Between the ASD Group and Controls Without Documented Neurodevelopmental Disorders

Secondary analyses comparing children with ASD (*n* = 23) with controls without documented neurodevelopmental disorders (*n* = 27) were restricted to SE measurements obtained with Retinomax, as Plusoptix measurements were not available for the control group. Because the groups differed in age, comparisons were performed using ANCOVA with age included as a continuous covariate. No evidence of a Group × Age interaction was found for any of the four outcomes (OD before cycloplegia: *p* = 0.506; OS before cycloplegia: *p* = 0.966; OD after cycloplegia: *p* = 0.706; OS after cycloplegia: *p* = 0.812), indicating that the homogeneity-of-regression-slopes assumption was not contradicted. The final models therefore included the main effects of Group and Age.

Before cycloplegia, age-adjusted mean SE was substantially lower in the ASD group than in the control group for both eyes. For OD, the adjusted mean was −2.36 D (95% CI: −3.05 to −1.67) in the ASD group and 1.75 D (95% CI: 1.10 to 2.39) in the control group, corresponding to an adjusted between-group difference of −4.10 D (F(1, 47) = 68.02, *p* < 0.001). For OS, the corresponding adjusted means were −2.42 D (95% CI: −3.20 to −1.64) and 1.71 D (95% CI: 0.99 to 2.44), respectively, with an adjusted difference of −4.13 D (F(1, 47) = 54.11, *p* < 0.001).

After cycloplegia, the adjusted mean SE remained lower in the ASD group than in the control group. For OD, adjusted means were −0.25 D (95% CI: −0.99 to 0.49) and 2.45 D (95% CI: 1.75 to 3.14), respectively, corresponding to an adjusted difference of −2.70 D (F(1, 47) = 25.44, *p* < 0.001). For OS, adjusted means were −0.25 D (95% CI: −0.99 to 0.49) in the ASD group and 2.35 D (95% CI: 1.66 to 3.04) in the control group, with an adjusted difference of −2.60 D (F(1, 47) = 23.81, *p* < 0.001) (Table 3).

## 4. Discussion

The principal finding of this pilot study was a systematic difference between refractive measurements obtained with Retinomax and Plusoptix in the same children with ASD. In the paired subgroup, Retinomax yielded substantially more myopic SE values than Plusoptix before cycloplegia, and significant inter-device differences in SE persisted after cycloplegia. Bland–Altman analysis confirmed a systematic negative bias of Retinomax relative to Plusoptix, which was greater before cycloplegia and decreased, but remained present, after cycloplegia. Although cycloplegia-induced SE shifts were numerically greater with Retinomax, the between-device differences in shift magnitude did not reach statistical significance after correction for multiple comparisons. Taken together, these findings indicate systematic device-dependent differences in refractive measurements and suggest that Retinomax and Plusoptix should not be regarded as directly interchangeable in this population, particularly under non-cycloplegic conditions.

Ophthalmic abnormalities, including refractive errors, strabismus, and amblyopia, are relatively common in children with ASD [7,8,11]. However, the reported prevalence and distribution of individual refractive errors vary considerably across studies [8,9,10]. This heterogeneity may reflect differences in study populations and age distributions, as well as methodological factors related to refractive assessment, including the use of cycloplegia and the measurement technique. These considerations are particularly relevant in children with ASD, in whom limited cooperation, sensory hypersensitivity, and difficulties in maintaining stable fixation may complicate objective refractive assessment. Consequently, differences between measurement methods may have important implications for the interpretation of refractive findings in this population.

Accommodation remains a plausible contributor to the observed inter-device differences. In pediatric refractive assessment, accommodative activity may produce a transient myopic shift in non-cycloplegic measurements, and instability of the accommodative response has been described particularly in younger children [20,21]. Factors such as sensory hypersensitivity or emotional reactivity may further complicate accommodative control during examination in children with ASD, although the relationship between these factors and refractive measurements remains insufficiently established [22]. In the present study, the magnitude of the Retinomax–Plusoptix bias decreased after cycloplegia, which is compatible with an accommodative contribution to the observed discrepancy. However, cycloplegia-induced SE shifts, although numerically greater with Retinomax, did not differ significantly between the devices after correction for multiple comparisons. Moreover, the study design did not allow accommodative effects to be separated from device-specific measurement variability. Therefore, the observed pre- to post-cycloplegic changes should be interpreted as cycloplegia-induced refractive shifts rather than as direct evidence of pseudomyopia.

Differences in measurement characteristics between the two instruments may also have contributed to the observed discrepancies. Plusoptix uses binocular photorefraction from a greater working distance, whereas Retinomax is a handheld autorefractor requiring measurement at close range. These differences in measurement conditions may influence accommodative behavior during refractive assessment. Previous studies have reported satisfactory performance of both instruments in pediatric populations, particularly under cycloplegic conditions [23]. However, examination conditions and the ability to maintain adequate fixation may be particularly relevant in children with ASD or other developmental disabilities [24,25,26]. In the present study, Bland–Altman analysis demonstrated that the systematic difference between the devices decreased after cycloplegia but did not disappear, with Retinomax measurements remaining more myopic than Plusoptix measurements. This persistence of inter-device bias after cycloplegia suggests that accommodation alone may not fully explain the observed differences and that device-specific measurement characteristics should also be considered.

Previous studies have reported considerable variability in the refractive profiles of children with ASD. Some studies have identified hyperopia as a frequent refractive finding [8,27,28], whereas others have reported a greater contribution of astigmatism or different distributions of refractive errors [29]. Differences in age, clinical characteristics, examination conditions, and refractive methodology may contribute to this heterogeneity. The present findings add a methodological perspective to these observations by demonstrating that refractive estimates may differ systematically according to the device used, particularly under non-cycloplegic conditions. Therefore, comparisons of refractive-error patterns across studies should take into account not only whether cycloplegia was performed but also the measurement technique and instrument used.

In the secondary analysis, Retinomax SE measurements also differed between children with ASD and controls without documented neurodevelopmental disorders after adjustment for age, both before and after cycloplegia. These findings should, however, be interpreted cautiously. The control group was older and had a relatively narrow age distribution, with limited age overlap between groups, and measurements in the control group were available only for Retinomax. Thus, although the ANCOVA accounted for age as a continuous covariate and the homogeneity-of-regression-slopes assumption was not contradicted, statistical adjustment cannot fully compensate for the lack of age matching. Accordingly, the ASD–control comparison should be regarded as secondary and does not establish that the observed refractive differences are specifically attributable to ASD.

Cyclopentolate is generally regarded as the gold standard for inducing cycloplegia in children; however, its use may be associated with systemic adverse effects, particularly involving the central nervous system [30,31]. These may include drowsiness [32], altered consciousness [33], and, in rare cases, seizures [34,35,36,37,38] or severe allergic reactions, including anaphylactic shock [39,40,41,42]. Such risks appear to be more pronounced in younger children and those with lower body mass index [33]. Children with ASD may also present with neurological comorbidities, including epilepsy and sleep disturbances [43,44]. Tropicamide is often better tolerated, and several studies indicate that it can achieve clinically acceptable cycloplegia in selected pediatric age groups [13,14,15,16,45,46,47]. Nevertheless, some degree of residual accommodation cannot be entirely excluded, and this may have influenced post-cycloplegic values in our study. Importantly, the systematic inter-device difference persisted after cycloplegia, although its magnitude was reduced. This suggests that residual accommodation alone may not fully account for the observed differences between the two devices.

Several limitations should be acknowledged. The overall sample size was small, and paired measurements with both devices were available for only 14 children with ASD. This limitation largely reflects the challenges of refractive assessment in children with ASD, who may exhibit anxiety, distress, limited cooperation, or difficulty maintaining fixation during examination. These factors may also have introduced selection bias, because children who successfully completed measurements with both devices may have represented a more cooperative subgroup of the ASD cohort. Consequently, the paired inter-device analyses and the Bland–Altman estimates should be interpreted cautiously and require confirmation in larger prospective samples.

A further limitation concerns the controls without documented neurodevelopmental disorders. The control group was established retrospectively and differed substantially in age from the ASD group. Although age was included as a continuous covariate in the ANCOVA models and no evidence of a Group × Age interaction was found, the limited overlap in age distributions between the groups restricts the extent to which statistical adjustment can compensate for the lack of age matching. Moreover, refractive assessment in the control group was performed using Retinomax only, as Plusoptix measurements were not available. Consequently, the ASD–control comparison was restricted to Retinomax measurements and should be regarded as a secondary analysis; it cannot determine whether the observed between-group differences are independent of device-specific measurement characteristics.

The retrospective design may also have introduced additional selection bias and limited control over other potentially relevant clinical and procedural factors. Furthermore, the measurement order was fixed, with Plusoptix always preceding Retinomax. Although this sequence reflected the clinical examination protocol, a potential order effect cannot be excluded and cannot be separated from the observed device effect. Finally, ASD severity and related behavioral characteristics were not formally incorporated into the statistical analyses. Therefore, the extent to which the present findings generalize across children with different levels of ASD severity, cooperation, or comorbidity remains uncertain.

Despite these limitations, the findings have practical implications. They suggest that non-cycloplegic autorefractometry results in children with ASD should be interpreted cautiously, with attention to both accommodative factors and device characteristics. Whenever feasible, cycloplegic assessment remains advisable in this population. Importantly, however, the systematic difference between Retinomax and Plusoptix was reduced but not eliminated after cycloplegia, indicating that cycloplegia alone does not ensure agreement between measurements obtained with different autorefractors. Further prospective studies with larger samples, standardized and randomized measurement protocols, and complete inter-device assessments are needed to determine the clinical significance of these differences.

From a clinical perspective, the present findings emphasize the importance of considering both cycloplegic status and the measurement method when interpreting refractive results in children with ASD. In children for whom cyclopentolate use may be less desirable because of concerns regarding tolerability or potential adverse effects, tropicamide may provide a clinically practical alternative for cycloplegic refraction. However, residual accommodation cannot be excluded, and the present results demonstrate that systematic inter-device differences may persist even after cycloplegia. Therefore, refractive measurements obtained with different autorefractors should not be assumed to be directly interchangeable, particularly when monitoring refractive status over time or comparing results obtained with different measurement protocols.

In summary, our results demonstrate systematic device-related differences in refractive measurements in children with ASD, particularly under non-cycloplegic conditions. Retinomax yielded more myopic SE values than Plusoptix before cycloplegia, and this inter-device difference was reduced but persisted after cycloplegia. These findings underscore the importance of controlling accommodation and considering device-specific measurement characteristics when interpreting refractive assessments in children with ASD. The persistence of inter-device differences after cycloplegia suggests that accommodation alone may not fully explain the observed discrepancies. Further prospective studies with larger samples, randomized measurement order, standardized cycloplegic protocols, and complete inter-device comparisons in both children with ASD and controls without documented neurodevelopmental disorders are needed to determine the relative contributions of accommodation, measurement technique, and clinical characteristics to refractive variability.

## Figures and Tables

**Table 1:** Paired comparison of refractive measurements obtained with Plusoptix and Retinomax before and after cycloplegia in children with autism spectrum disorder (*n* = 14).

Parameter	Plusoptix, Mean ± SD (D)	Retinomax, Mean ± SD (D)	*p*	*r*_rb_
Before cycloplegia				
OD Dsph	1.30 ± 0.91	−0.80 ± 1.47	0.0010	1.000
OD Dcyl	−0.66 ± 0.74	−0.95 ± 0.68	0.0018	0.943
OD SE	0.91 ± 0.67	−1.21 ± 1.25	0.0010	1.000
OS Dsph	1.14 ± 0.64	−0.85 ± 1.25	0.0012	0.981
OS Dcyl	−0.61 ± 0.49	−0.85 ± 0.59	0.0022	0.924
OS SE	0.77 ± 0.51	−1.22 ± 1.15	0.0012	0.981
After cycloplegia				
OD Dsph	2.43 ± 0.85	0.93 ± 1.14	0.0010	1.000
OD Dcyl	−0.76 ± 0.66	−0.73 ± 0.69	0.551	−0.181
OD SE	2.00 ± 0.63	0.63 ± 0.94	<0.001	1.000
OS Dsph	2.34 ± 0.82	0.77 ± 1.06	0.0010	1.000
OS Dcyl	−0.63 ± 0.54	−0.76 ± 0.60	0.035	0.638
OS SE	1.98 ± 0.63	0.46 ± 0.96	<0.001	1.000

Data are presented as mean ± standard deviation (SD), in diopters (D). Paired comparisons between Plusoptix and Retinomax were performed using the Wilcoxon signed-rank test. Effect sizes are reported as matched-pairs rank-biserial correlations (r_rb_), calculated for Plusoptix minus Retinomax. Positive values indicate higher measurements with Plusoptix. After Bonferroni correction for 12 comparisons, statistical significance was set at α = 0.0042. OD—right eye; OS—left eye; Dsph—spherical diopters; Dcyl—cylindrical diopters; SE—spherical equivalent.

**Table 2:** Comparison of cycloplegia-induced refractive shifts between Plusoptix and Retinomax in children with autism spectrum disorder (*n* = 14).

Parameter	Plusoptix, Mean ± SD (D)	Retinomax, Mean ± SD (D)	*p*	*r*_rb_
OD Dsph	1.13 ± 0.81	1.73 ± 1.11	0.084	−0.524
OD Dcyl	−0.09 ± 0.41	0.21 ± 0.29	0.023	−0.744
OD SE	1.09 ± 0.71	1.84 ± 1.11	0.058	−0.581
OS Dsph	1.21 ± 0.84	1.62 ± 1.13	0.367	−0.295
OS Dcyl	−0.01 ± 0.34	0.09 ± 0.35	0.182	−0.436
OS SE	1.20 ± 0.77	1.67 ± 1.19	0.272	−0.333

Data are presented as mean ± standard deviation (SD), in diopters (D). Cycloplegia-induced shifts were calculated as post-cycloplegic minus pre-cycloplegic values. Between-device comparisons were performed using the Wilcoxon signed-rank test. Effect sizes are expressed as matched-pairs rank-biserial correlations (r_rb_), calculated for Plusoptix minus Retinomax. Negative values indicate higher shift values with Retinomax than with Plusoptix. After Bonferroni correction for six comparisons, statistical significance was set at α = 0.0083. OD—right eye; OS—left eye; Dsph—spherical diopters; Dcyl—cylindrical diopters; SE—spherical equivalent.

**Table 3:** Age-adjusted comparison of spherical equivalent measured with Retinomax between children with ASD and controls without documented neurodevelopmental disorders before and after cycloplegia.

SE Outcome	Group × Age *p*	ASD Adjusted Mean (95% CI), D	Control Adjusted Mean (95% CI), D	Adjusted Difference (ASD—Control), D	Group F(1, 47)	*p*
OD before cycloplegia	0.506	−2.36 (−3.05 to −1.67)	1.75 (1.10 to 2.39)	−4.10	68.02	<0.001
OS before cycloplegia	0.966	−2.42 (−3.20 to −1.64)	1.71 (0.99 to 2.44)	−4.13	54.11	<0.001
OD after cycloplegia	0.706	−0.25 (−0.99 to 0.49)	2.45 (1.75 to 3.14)	−2.70	25.44	<0.001
OS after cycloplegia	0.812	−0.25 (−0.99 to 0.49)	2.35 (1.66 to 3.04)	−2.60	23.81	<0.001

Adjusted means are estimated marginal means from analysis of covariance (ANCOVA) models including Group and Age. The Group × Age interaction was tested separately before fitting the final main-effects models. CI—confidence interval; ASD—autism spectrum disorder; OD—right eye; OS—left eye; SE—spherical equivalent; D—diopters.

## Data Availability

All data generated or analyzed during this study are included in this published article and its Supplementary Information Files.

## Supplementary Materials

The following supporting information can be downloaded at: https://www.mdpi.com/article/10.3390/jcm15207889/s1, Supplementary Table S1: Refraction results obtained using the Retinomax device in children with autism spectrum disorder before and after cycloplegia, prior to regression adjustment. Supplementary Table S2: Refraction results obtained using the Plusoptix device in children with autism spectrum disorder before and after cycloplegia, prior to regression adjustment. Supplementary Table S3: Completed STROBE reporting checklist.

## Abbreviations

The following abbreviations are used in this manuscript:

ANCOVA	Analysis of covariance
ASD	Autism Spectrum Disorder
CI	Confidence interval
D	Diopter
Dcyl	Cylindrical diopters
DSM-5	Diagnostic and Statistical Manual of Mental Disorders, Fifth Edition
Dsph	Spherical diopters
ICD-10	International Classification of Diseases, 10th Revision
LoA	Limits of agreement
n	Number of subjects
OD	Oculus Dexter (right eye)
OS	Oculus Sinister (left eye)
p	*p*-value (probability value)
r_rb_	matched-pairs rank-biserial correlation
SD	Standard deviation
SE	Spherical equivalent
