# Supplementary material for: Variability in Refractive Measurements in Children with Autism Spectrum Disorder Under Cycloplegic and Non-Cycloplegic Conditions: Influence of Measurement Method
Source: J Clin Med. 2026 Oct 13;15(20):7889. doi: 10.3390/jcm15207889 (PMC13705275; doi:10.3390/jcm15207889)
Supplement: Supplementary file 1 [file jcm-15-07889-s001.zip › jcm-4525408-supplementary.pdf]

Child	Age (in years)	Before OD			Before OS			After OD			After OS			Difference in D		Difference in Sph Eq	
		Dsph	Dcyl	Sph Eq	Dsph	Dcyl	Sph Eq	Dsph	Dcyl	Sph Eq	Dsph	Dcyl	Sph Eq	Before/ after OD	Before/ after OS	Before/ after OD	Before/ after OS
**1**	5	-1.50	-0.50	-1.75	-1.00	-0.25	-1.13	2.25	-0.50	2.00	1.50	-0.25	1.38	3.75/ 0.00	2.50/ 0.00	3.75	2.50
**2**	8	3.00	-2.00	2.00	2.25	-1.00	1.75	3.00	-2.00	2.00	2.50	-1.25	1.88	0.00/ 0.00	0.25/ -0.25	0.00	0.13
**3**	12	-6.50	-1.25	-7.13	-5.50	-1.75	-6.38	0.72	-0.51	0.47	0.72	-0.63	0.41	7.22/ 0.74	6.22/ 1.12	7.59	6.78
**4**	12	-3.25	0.00	-3.25	-2.50	-0.50	-2.75	0.25	0.00	0.25	-1.00	-0.50	-1.25	3.50/ 0.00	1.50/ 0.00	3.50	1.50
**5**	12	-9.75	-1.00	-10.25	-10.25	-1.50	-11.00	-10.00	-1.00	-10.50	-7.00	-2.50	-8.25	-0.25/ 0.00	3.25/ 1.00	-0.25	2.75
**6**	7	1.25	-2.75	-0.13	1.00	-2.75	-0.38	3.00	-2.50	1.75	2.75	-2.50	1.50	1.75/ 0.25	1.75/ 0.25	1.88	1.88
**7**	3	1.00	-1.50	0.25	0.25	-1.25	-0.38	2.75	-1.00	2.25	2.00	-1.00	1.50	1.75/ 0.50	1.75/ 0.25	2.00	1.88
**8**	3.5	-1.25	-0.75	-1.62	-1.25	-0.75	-1.65	-1.25	-0.50	-1.50	-1.00	-1.25	-1.63	0.40/ 0.16	-0.27/ -0.49	0.12	0.02
**9**	6	-2.75	-1.00	-3.25	-3.25	-0.75	-3.63	0.75	-1.00	0.25	2.00	-0.75	1.63	3.50/ 0.00	5.75/ 0.00	3.50	5.25
**10**	3	-1.25	-0.75	-1.62	-1.25	-0.75	-1.65	1.75	-0.25	1.63	1.50	-0.50	1.25	3.04/ 0.41	2.77/ 0.26	3.25	2.90
**11**	7	-1.25	-0.75	-1.62	-1.25	-0.75	-1.65	0.25	-1.75	-0.63	0.25	-1.00	-0.25	1.54/ 1.09	1.52/ -0.24	1.00	1.40
**12**	5	-1.25	-0.75	-1.62	-1.25	-0.75	-1.65	0.00	0.00	0.00	0.25	0.00	0.25	1.29/ 0.66	1.52/ 0.76	1.62	1.90
**13**	3	-1.25	-0.75	-1.62	-1.25	-0.75	-1.65	1.75	0.00	1.75	1.25	-0.25	1.13	3.04/ 0.66	2.52/ 0.51	3.37	2.78
**14**	7.5	-3.25	0.00	-3.25	-4.25	-0.50	-4.50	0.75	-0.25	0.63	0.00	0.00	0.00	4.00/ -0.25	4.25/ 0.50	3.88	4.50
**15**	4.5	-0.50	-0.25	-0.63	0.00	-0.50	-0.25	0.72	-0.51	0.47	0.72	-0.63	0.41	1.22/ -0.26	0.72/ -0.13	1.09	0.66
**16**	6.5	-1.25	-0.75	-1.62	-1.25	-0.75	-1.65	1.25	-1.00	0.75	2.00	-1.25	1.38	2.54/ -0.34	3.27/ -0.49	2.37	3.03
**17**	6	-0.75	-0.25	-0.88	-0.75	-0.25	-0.88	0.72	-0.51	0.47	0.72	-0.63	0.41	1.47/ -0.26	1.47/ -0.38	1.34	1.28
**18**	5	-0.25	-0.25	-0.38	-0.75	0.00	-0.75	0.75	-0.25	0.63	0.50	-0.25	0.38	1.00/ 0.00	1.25/ -0.25	1.00	1.13
**19**	2.5	-1.25	-0.75	-1.62	-1.25	-0.75	-1.65	1.00	0.00	1.00	0.75	0.00	0.75	2.29/ 0.66	2.02/ 0.76	2.62	2.40
**20**	8	-1.00	-0.50	-1.25	-0.75	-0.25	-0.88	1.00	-0.50	0.75	1.00	-0.50	0.75	2.00/ 0.00	1.75/ -0.25	2.00	1.63

**21**	8	-1.25	-0.75	-1.62	-1.25	-0.75	-1.65	0.25	-0.25	0.13	0.25	-0.25	0.13	1.54/ 0.41	1.52/ 0.51	1.75	1.78
**22**	8	-1.25	-0.75	-1.62	-1.25	-0.75	-1.65	1.50	-0.25	1.38	1.50	-0.25	1.38	2.79/ 0.41	2.77/ 0.51	3.00	3.03
**23**	8	0.25	-0.25	0.13	0.50	-1.25	-0.13	0.25	0.00	0.25	0.25	-1.00	-0.25	0.00/ 0.25	-0.25/ 0.25	0.13	-0.13
**Average**		**-1.53**	**-0.75**	**-1.99**	**-1.57**	**-0.80**	**-1.89**	**0.61**	**-0.63**	**0.20**	**0.58**	**-0.68**	**0.31**	**2.10/ 0.23**	**2.05/ 0.18**	**2.19**	**2.10**
**SD**		**2.42**	**0.61**	**2.62**	**2.49**	**0.54**	**2.43**	**2.44**	**0.66**	**2.51**	**1.89**	**0.74**	**2.30**	**1.80/ 0.36**	**1.57/ 0.51**	**1.77**	**1.61**

D – diopters; Dsph – spherical diopters; Dcyl – cylindrical diopters; Sph Eq – spherical equivalents; OD – right eye; OS – left eye; SD –standard deviation

Supplementary Table S1. Refraction results obtained using the Retinomax device in children with autism spectrum disorder, before and after cycloplegia, prior to regression adjustment.

Child	Age (in years)	Before OD			Before OS			After OD			After OS			Difference in D		Difference in Sph Eq	
		**Dsph**	**Dcyl**	**Sph Eq**	**Dsph**	**Dcyl**	**Sph Eq**	**Dsph**	**Dcyl**	**Sph Eq**	**Dsph**	**Dcyl**	**Sph Eq**	**Before/ after OD**	**Before/ after OS**	**Before/ after OD**	**Before/ after OS**
**1**	5	0.50	-0.25	0.38	0.50	-0.25	0.38	2.58	-0.86	2.14	2.17	-0.73	1.81	2.08/-0.61	1.67/-0.48	1.77	1.44
**2**	8	2.75	-1.75	1.88	1.75	-1.25	1.13	3.50	-1.00	3.00	3.25	-1.50	2.50	0.75/0.75	1.50/-0.25	1.13	1.38
**4**	12	0.80	0.25	0.93	0.75	-0.50	0.50	2.25	-0.25	2.13	1.75	-0.50	1.50	1.45/-0.50	1.00/0.00	1.20	1.00
**6**	7	2.50	-2.75	1.13	2.00	-2.25	0.88	4.00	-2.75	2.63	4.25	-2.50	3.00	1.50/0.00	2.25/-0.25	1.50	2.13
**7**	3	2.75	-2.00	1.75	1.50	-1.25	0.88	3.75	-2.25	2.63	3.00	-0.75	2.63	1.00/-0.25	1.50/0.50	0.88	1.75
**8**	3.5	0.75	-0.50	0.50	0.50	-0.50	0.25	0.50	-0.25	0.38	0.75	-0.25	0.63	-0.25/0.25	0.25/0.25	-0.13	0.38
**9**	6	0.25	-0.50	0.00	0.75	-0.50	0.50	1.75	-0.50	1.50	3.25	-1.00	2.75	1.50/0.00	2.50/-0.50	1.50	2.25
**10**	3	2.75	-0.50	2.50	2.75	-0.75	2.38	2.58	-0.86	2.14	2.17	-0.73	1.81	-0.18/-0.36	-0.58/0.03	-0.36	-0.57
**12**	5	1.00	-0.75	0.72	1.0	-0.75	0.61	1.25	-0.25	1.13	1.25	-0.25	1.13	0.21/0,4	0.33/0.38	0.41	0.52
**15**	4.5	0.50	-0.25	0.38	0.50	-0.25	0.38	2.58	-0.86	2.14	2.17	-0.73	1.81	2.08/-0.61	1.67/-0.48	1.77	1.44
**18**	5	0.00	-0.25	-0.13	0.25	-0.25	0.13	2.75	-1.00	2.25	2.75	-0.50	2.50	2.75/-0.75	2.50/-0.25	2.38	2.38
**19**	2.5	1.00	-0.75	0.72	1.00	-0.75	0.61	3.00	-0.50	2.75	3.00	-0.25	2.88	1.96/0.15	2.08/0.38	2.04	2.27
**20**	8	1.00	-0.75	0.72	1.00	-0.75	0.61	2.50	-1.00	2.00	2.25	-0.75	1.88	1.46/-0.35	1.33/-0.13	1.29	1.27
**21**	8	1.75	-1.00	0.81	1.50	-1.00	1.00	2.58	-0.86	2.14	2.17	-0.73	1.81	1.27/0.14	0.67/0.28	1.33	0.81
**Average**		**1.31**	**-0.91**	**0.88**	**1.05**	**-0.77**	**0.77**	**2.68**	**-0.94**	**2.20**	**2.57**	**-0.80**	**2.03**	**1.18/-0.14**	**1.26/-0.04**	**1.19**	**1.32**
**SD**		**1.04**	**0.76**	**0.66**	**0.62**	**0.54**	**0.52**	**0.92**	**0.65**	**0.62**	**0.88**	**0.56**	**0.65**	**0.91/0.46**	**0.82/0.34**	**0.86**	**0.83**

D – diopters; Dsph – spherical diopters; Dcyl – cylindrical diopters; Sph Eq – spherical equivalents; OD – right eye; OS – left eye; SD –standard deviation

Supplementary Table S2. Refraction results obtained using the Plusoptix device in children with autism spectrum disorder, before and after cycloplegia, prior to regression adjustment.

Supplementary Table S3.

STROBE reporting checklist

Cross sectional studies

Manuscript ID jcm-4525408

Variability in Refractive Measurements in Children with Autism Spectrum Disorder under Cycloplegic and Non-Cycloplegic Conditions: Influence of Measurement Method

Page and line references correspond to the 17-page manuscript PDF dated 29 September 2026. STROBE item numbers are retained; reporting elements are abbreviated. "Not reported" identifies a reporting gap; "not applicable" identifies an irrelevant item.

Title abstract introduction and methods

Item	STROBE reporting element	Manuscript location and response
**1(a)**	Identify the study design.	p. 1, Abstract, lines 26-30; p. 3, Materials, lines 114-123. Described as a retrospective pilot study and a retrospective observational review of medical records.
**1(b)**	Summarize methods and findings in a balanced abstract.	pp. 1-2, Abstract, lines 24-57. Reports the samples, paired analyses, agreement assessment, secondary age-adjusted comparisons, principal results and cautious conclusions.
**2**	Explain the scientific context and rationale.	pp. 2-3, Introduction, lines 62-104. Discusses ASD, difficulties in pediatric refraction, accommodation and potential device-dependent differences.
**3**	Specify objectives and prespecified hypotheses, if any.	p. 3, Introduction, lines 105-112. Primary and secondary objectives are specified; no prespecified directional hypotheses are stated.
**4**	Describe the central design features early.	pp. 3-4, Materials, lines 114-138; control selection, lines 174-185. Retrospective record review with paired device comparisons and a secondary comparison group.
**5**	Identify the setting, locations and relevant dates.	p. 3, Materials, lines 114-123; p. 4, lines 174-185. Examinations at the Pediatric Ward, Second Department of Ophthalmology, University Clinical Hospital No. 2, Pomeranian Medical University in Szczecin, June 2020-May 2021; controls from the same center and period.
**6(a)**	Explain eligibility, participant sources and selection.	pp. 3-4, Materials, lines 114-138 and 174-185. ASD diagnosis documented before examination; availability of pre- and post-cycloplegic Retinomax measurements required. Controls had no documented ASD or other neurodevelopmental disorder. All available eligible records were included.
**7**	Define outcomes, predictors, confounders and diagnostic criteria.	pp. 3-6, lines 124-135, 147-150, 188-189, 199-243. Defines documented ASD using ICD-10 F84, refractive parameters, SE, diagnostic group, age, device differences and cycloplegia-induced shifts.
**8**	Describe data sources, measurements and comparability across groups.	pp. 3-4, lines 124-185. Archived records, clinical assessments, devices, fixed measurement order, tropicamide protocol and standardized examination conditions are described. Both groups had Retinomax measurements; Plusoptix was available only in the ASD paired subgroup.

Methods continued

Item	STROBE reporting element	Manuscript location and response
**9**	Describe steps taken to reduce potential bias.	pp. 4-5, lines 160-173 and 199-215: fixed examination protocol, one ophthalmologist, consistent conditions and age adjustment. pp. 11-12, lines 411-420 and 435-462: residual age confounding, selection bias, fixed-order effects and limited generalisability are discussed.
**10**	Explain the basis for the sample size.	pp. 3-4, lines 133-138 and 174-182. All available eligible records were included; no a priori sample-size calculation because the study was retrospective and exploratory. Samples: 23 ASD participants, 14 with paired devices, and 27 controls.
**11**	Explain treatment of numerical variables and any grouping.	pp. 4-6, Statistical Analysis, lines 186-247. Refractive values analyzed continuously in diopters; age entered as a continuous covariate. Means and SDs, SE calculation, paired differences, refractive shifts and agreement limits are defined. No continuous outcomes were categorized for the principal analyses.
**12(a)**	Describe statistical procedures and confounding control.	pp. 5-6, lines 190-247. Shapiro-Wilk, Wilcoxon signed-rank tests, Bonferroni correction, matched-pairs rank-biserial correlations, Bland-Altman analysis and age-adjusted ANCOVA; separate analyses by eye and cycloplegic condition. Software and two-sided significance thresholds are specified.
**12(b)**	Explain subgroup and interaction analyses.	pp. 4-6, lines 151-159, 199-215 and 227-243. Device comparisons use the 14 complete paired observations; secondary ANCOVA uses all 23 ASD participants and 27 controls. Group by Age interactions are tested before fitting the final models.
**12(c)**	Explain the handling of missing observations.	p. 4, lines 151-159 and 174-185. No imputation; device comparisons restricted to complete paired observations. Nine ASD participants lacked complete Plusoptix measurements because of limited cooperation; controls lacked Plusoptix measurements. p. 4, lines 143-146: incomplete clinical assessments are acknowledged.
**12(d)**	Describe adjustments for the sampling scheme, when relevant.	Not applicable to survey-weighted or complex-sampling analyses. All available eligible clinical records from the stated period were included (pp. 3-4, lines 114-138 and 174-182); no complex sampling scheme is described.
**12(e)**	Describe sensitivity analyses.	No sensitivity analyses are reported. The Group by Age interaction tests are model-assumption checks, rather than sensitivity analyses (p. 5, lines 209-215).

Results

Item	STROBE reporting element	Manuscript location and response
**13(a)**	Give participant counts at each selection and analysis stage.	Partially reported. pp. 3-4, lines 124-138 and 151-159; p. 6, lines 255-264; p. 9, lines 318-320. Reports 23 ASD participants, 14 complete paired device examinations and 27 controls. Counts of records initially screened or excluded before final inclusion are not reported.
**13(b)**	Explain reasons for nonparticipation or incomplete assessment.	p. 4, lines 143-159: limited cooperation prevented complete Plusoptix measurements in nine children and limited clinical assessments. p. 6, lines 255-257: visual acuity assessed in 15 children. Reasons and counts for exclusions before final inclusion are not reported.
**13(c)**	Consider a participant flow diagram.	No participant flow diagram is included. Included and analyzed sample sizes are described in the text (pp. 3-4, 6 and 9; see item 13(a)).
**14(a)**	Describe participant characteristics and potential confounders by group.	Partially reported. pp. 3-4, lines 124-146: ASD age, sex, gestational age and birth weight; p. 6, lines 255-261: recorded ophthalmologic findings. p. 4, line 174: control mean age 8.58 years. Control sex distribution, age SD and age range are not reported.
**14(b)**	Give missing-data counts for relevant variables.	Partially reported. p. 4, lines 143-159: nine of 23 lacked complete Plusoptix measurements; visual acuity available in 15 of 23. Controls had no Plusoptix measurements (lines 174-185). Exact counts for incomplete anterior-segment/fundus assessments and a comprehensive variable-level missing-data summary are not reported.
**15**	Present outcome counts or summary statistics.	pp. 6-9, Results, lines 255-347. Recorded ocular findings and visual acuity; Table 1: paired device measurements; Table 2: refractive shifts; Figure 1 and lines 301-307: SE bias and limits of agreement; Table 3: age-adjusted group comparisons.
**16(a)**	Present crude and adjusted estimates, precision and adjustment rationale.	Partially reported. Tables 1-2 (pp. 6-7) give paired means, SDs, p values and effect sizes; Figure 1 (p. 8) gives agreement estimates. Table 3 (p. 9) reports age-adjusted group means with 95% CIs and adjusted differences. Age adjustment is justified on p. 5, lines 199-215. CIs for adjusted differences and unadjusted ASD-control estimates are not reported in the main text.
**16(b)**	Give cutoffs for categorized continuous measures.	Not applicable. Principal refractive outcomes and age were analyzed as continuous variables (pp. 4-6, lines 147-150 and 186-243).
**16(c)**	Translate relative into absolute risk, when meaningful.	Not applicable. This measurement-comparison study does not estimate relative risks or time-dependent event risks.
**17**	Report subgroup, interaction and other additional analyses.	p. 7, Table 2 and lines 283-298: paired comparison of refractive shifts. p. 9, lines 318-347 and Table 3: secondary age-adjusted group comparisons and Group by Age interaction results. No sensitivity-analysis results are reported.

Discussion and other information

Item	STROBE reporting element	Manuscript location and response
**18**	Relate the principal findings to the objectives.	p. 10, Discussion, lines 350-361; p. 13, lines 483-495. Summarizes systematic device differences, persistence after cycloplegia and nonsignificant differences in shift magnitude after multiplicity correction.
**19**	Discuss limitations, imprecision and potential bias direction and magnitude.	pp. 10-13, lines 372-400, 411-462 and 477-495. Discusses accommodation, device effects, small sample, cooperative-subgroup selection, age imbalance, retrospective selection, absent control Plusoptix measurements, fixed device order and unmeasured ASD severity. The direction and magnitude of residual biases are not quantified.
**20**	Interpret cautiously alongside limitations, multiplicity and other evidence.	pp. 10-13, Discussion, lines 350-495. Considers prior studies and multiple-testing correction, avoids diagnosing pseudomyopia from refractive shifts, and treats ASD-control comparisons as secondary without attributing differences specifically to ASD.
**21**	Discuss applicability beyond the study population.	p. 12, lines 435-462, particularly lines 439-443 and 459-462; p. 13, lines 483-495. Generalisability across ASD severity, cooperation and comorbidity is uncertain; larger prospective samples and randomized measurement order are proposed.
**22**	Disclose funding and sponsor involvement, including original data collection.	p. 13, Funding, line 503: the research received no external funding. No external funder role is applicable to the reported study. Funding of the original clinical data collection is not separately specified.

Checklist sources

STROBE Initiative. Checklist for cross-sectional studies and combined checklist for observational studies. Group-specific information is provided where available.

https://www.strobe-statement.org/download/strobe-checklist-cross-sectional-studies-pdf

https://www.strobe-statement.org/download/strobe-checklist-cohort-case-control-and-cross-sectional-studies-combined
